# Supplementary material for: (3α,5α)3-Hydroxypregnan-20-one (3α,5α-THP) Regulation of the HPA Axis in the Context of Different Stressors and Sex
Source: Biomolecules. 2022 Aug 18;12(8):1134. doi: 10.3390/biom12081134 (PMC9406198; doi:10.3390/biom12081134)
Supplement: Supplementary file 1 [file biomolecules-12-01134-s001.zip › biomolecules-1832615-supplementary.pdf]

Table S1. Characteristics of primary antibodies used in immunoblotting.

Target	Commercial supplier	Cat No #	Clonality	Host	Dilution
CRF	Immunostar	20084	Polyclonal	Rabbit	1:1000
CRFBP	Santa Cruz Biotechnology	Sc-365426	Monoclonal	Mouse	1:500
CRFR1	Novus Biologicals	NBP1-00175	Polyclonal	Goat	1:1000
GR	Abcam	EPR19621	Monoclonal	Rabbit	1:1000
POMC	Novus Biologicals	NB100-1533	Polyclonal	Goat	1:1000
B-Actin	Novus Biologicals	NB600-501	Monoclonal	Mouse	1:5000
